# Dietary Fish Oil Can Change Sperm Parameters and Fatty Acid Profiles of Ram Sperm during Oil Consumption Period and after Removal of Oil Source

**Published:** 2014-10-04

**Authors:** AliReza Alizadeh, Vahid Esmaeili, Abdolhossein Shahverdi, Ladan Rashidi

**Affiliations:** 1 Department of Animal Science, Saveh Branch, Islamic Azad University, Saveh, Iran; 2 Department of Embryology at Reproductive Biomedicine Research Center, Royan Institute for Reproductive Biomedicine, ACECR, Tehran, Iran; 3 Institute of Standard and Industrial Research of Iran (ISIRI), Karaj, Iran

**Keywords:** Fish Oil, Ovine, Spermatozoa

## Abstract

**Objective:**

The effects of dietary fish oil on semen quality and sperm fatty acid profiles during consumption of n-3 fatty acids as well as the persistency of fatty acids in ram’s sperm after removing dietary oil from the diet were investigated.

**Materials and Methods:**

In this experimental study, we randomly assigned 9 Zandi rams to two groups (isoenergetic and isonitrogenous diets): control (CTR; n=5) and fish oil (FO; n=4) for 70 days with a constant level of vitamin E in both groups. Semen was collected at the first week and at the last week of the feeding period (phase 1). After the feeding period, all rams were fed a conventional diet and semen samples were collected one and two months after removal of FO (phase 2). The sperm parameters and fatty acid profiles were measured by computer assisted semen analyzer (CASA) and gas chromatography (GC), respectively. The completely randomized design was used and data were analyzed with SPSS version 16.

**Results:**

Dietary FO had significant positive effects on all sperm quality and quantity parameters compared with the CTR during the feeding period (p<0.05). The positive effects of FO on sperm concentration and total sperm output were observed at one and two months after removal of FO (p<0.05), whereas other sperm parameters were unaffected. Before feeding, C14 (myristic acid), C16 (palmitic acid), C18 (stearic acid), C18:1 (oleic acid) and C22:6 (docosahexaenoic acid: DHA) were the primary sperm FA. FO in the diet increased sperm DHA, C14:0 and C18:0 during the feeding period (p<0.05).

**Conclusion:**

The present study showed not only manipulation of ram sperm fatty acid profiles by dietary FO and sperm parameters during the feeding period, but also the persistency of unique effects of dietary omega-3 fatty acids up to two months following its removal from the diet. Also, we recommend that sperm fatty acid profiles should be comprehensively analyzed and monitored.

## Introduction

Testicular cells and spermatozoa contain high amounts of polyunsaturated fatty acids (PUFA) and dietary fatty acids can manipulate fatty acid profiles of reproductive tissues. Although key roles of sperm fatty acid profiles in fertility have been confirmed, limited and inconsistent data are available on the relation between several dietary fatty acids and male reproduction. Some omega-3 and omega-6 PUFA (with 22 carbon) such as docosahexaenoic acid (DHA, C22:6 n-3) for human and ruminants, docosapantaenoic acid (DPA, C22:5 n-6) for boars, rodents and rabbits, and docosatetraenoic acid (DTA, C22:4, n-6) for domestic birds are recognized as major elements in spermatozoa phospholipids (1).

Dietary fatty acids may influence fatty acid profiles in several organs or products in humans and animals. Fish oil is a major source of DHA and eicosapentaenoic acid (EPA, C20:5 n-3). It improves sperm quality and quantity with vitamin E supplemented by more than five weeks consumption in rams (2) as well as humans (3).

Although the changes in sperm fatty acid profiles by fatty acid supplementation have been reported in almost all studies on humans (3, 4), boars (5), roosters (6) and rams (2, 7, 8), this manipulation in some cases did not coincide with improved sperm parameters (4, 5). Unsaturated fatty acid supplementation can even disturb ram sperm parameters when used without vitamin E supplementation (8). Very few studies have been carried out on sperm fatty acid profiles following the removal of dietary fatty acids (9, 10), even though this may affect future responses in humans and animals.

On the other hand, major and minor fatty acids in membranes can affect fluidity of biological compounds such as milk fat triglycerides and sperm phospholipids. Dairy researchers pinpoint the critical role of some minor fatty acids in milk fat fluidity (11). Despite the bulk of studies on milk fatty acid profiles in dairy ruminants, the fatty acid profiles in sperm have not been tested by researchers and only a few studies have been reported on some major fatty acids (1, 12). Establishing a negative correlation between increased saturated fatty acids (12) or trans fatty acids (13) and normal sperm parameters can reveal the significance of comprehensive reporting of fatty acid profiles. The negative correlation between decreased spermatozoa total lipid, increased saturated fatty acid content and sperm parameters has previously been reported in infertile boars (14). Therefore, all fatty acids in profiles can influence sperm parameters and some indices such as the desaturase index (monounsaturated fatty acids: saturated fatty acids ratio) can be used for comparison.

In an earlier study (7), we demonstrated that after 35 days despite the removal of dietary fatty acid sources, there were significantly different sperm fatty acid profiles observed between rams fed by saturated, omega- 6 and omega-3 fatty acids. However, fatty acid profiles at the commencement of the study and sperm parameters were not shown. Therefore, the aims of the present study were to compare sperm fatty acids profiles by fish oil consumption with a control diet and to determine whether fatty acids persist one and two months after removal of FO from the diet. The study also aimed to investigate changes in seminal plasma and sperm parameters during these periods.

## Materials and Methods

All chemical reagents were obtained from Sigma (St. Louis, MO, USA) unless otherwise indicated. This study received approval from the Ethics Committee of Royan Institute. This was an original research on sperm fatty acid profiles performed as a complete randomized design.

### Experimental location

This study was undertaken from August 2011 to January 2012 at the Zandi’s breeding station (Pishva, Varamin, Iran) and Royan Institute (Tehran, Iran).

### Experimental animals

A total of 9 Zandi rams (average age: 2.5 years and 70 ± 7.6 kg average body weights) were randomized assigned to two groups: control (CTR, n=5) and FO (n=4). Different pens separated the groups according to the type of treatment. Rams were allocated to the experimental groups based on principal component analysis of sperm quality prior to the commencement of the trial in order to balance both high and low quality semen producing rams between the groups.

### Experimental diet

Both groups were fed a constant level of vitamin E and isoenergetic and isonitrogenous rations (Table 1) that had metabolizable energy (ME) of 2.95 Mcal/kg and 12% crude protein (CP) in dry matter basis. Rations were given in two equal quantities per day. Rams were allowed to walk freely. Fish oil was provided by Kilka Fish Oil (Khazar Fish Powder Co., Kiashahr Port, Iran). The fatty acid profile of the fish oil was analyzed by gas chromatography (GC) in the Institute of Standard and Industrial Research of Iran (ISIRI), Karaj, Iran (Table 2). Rams adapted to the oil consumption within three weeks and after this period each ram was able to consume 35 g/d of fish oil. The ram’s body weights were recorded at the first and the final week of phase 1.

**Table 1:** Ingredients of the diets [% of dry matter (DM)]


Ingredients	CTR	Fish oil

**Alfalfa hay**	48	50
**Wheat straw**	15.5	27
**Barley**	30	14
**Molasses**	5	5
**Mineral and vitamin supplement**	1	1
Vitamin E*	0.5	0.5
**Fish oil**	-	2.5


*; Contains 5500 IU/kg vitamin E as provided by Vetaque Co. (Tehran, Iran).

**Table 2:** Fatty acid profiles of Iranian fish oil (% of total fatty acids)


Fatty acids	Concentration (%)

C12:0	0.06
C14:0	3.5
C14:1	0.6
C15:0	0.8
C15:1	0.2
C16:0	20.2
C16:1	6.9
C17:0	1.7
C17:1	0.9
C18:0	4.1
C18:1t	0.15
C18:1c	32
C18:2t	0.15
C18:2c	3
C18:3 gamma	0.17
C20:0	0.26
C18:3 alpha	1.8
C18:4	2.4
C20:1	0.4
C20:2	0.3
C20:3	0.06
C22:0	0.3
C20:4	0.6
C22:1	0.5
C20:5 (EPA)*	5.6
C24:0	0.22
C24:1	1.4
C22:5	0.35
C22:6 (DHA) *	10.6


### Experimental design

This experiment was carried out in two phases. In the first phase the rams received experimental diets for eight weeks. After this feeding period, all rams were fed a conventional diet for two months (phase 2).

### Semen analysis

Semen samples were collected from each ram using an artificial vagina (AV). The sampling times were repeated for two days during one week. The four sampling weeks included: the commencement of the study (week one), eight weeks after fish oil consumption, one month after removing dietary fish oil, and two months after removal of dietary fish oil. Semen samples were maintained at 4˚C in an INRA96 extender (INRA Co., France) and immediately transferred from the farm to the laboratory for evaluation. Semen volume was measured using conical graded tubes. Sperm count and motility were analyzed by a computer-assisted sperm analyzer (CASA). The CASA system consists of a phase contrast microscope (Eclipse E-200, Nikon Co., Tokyo, Japan) with a heat plate equipped with Sperm Class Analyzer® software (SCA, Full Research Version 5.1, Microptic Co., Barcelona, Spain). For analysis, 5 μl of sperm samples were placed in a 20 μm standard count analysis chamber (Leja, Nieuw Vennep Co., Luzernestraat Nieuw Vennep, The Netherlands) and observed with a Nikon microscope 10×0.25 negative phase contrast field Ph1 BM with a green filter (10). Several fields of view were captured and at least 1000 spermatozoa were counted in each analysis (the system was set up with analysis of ovine values).

The viability of sperm in the sample was assessed by eosin-nigrosin stain. The sperm smears were prepared by mixing a drop of semen with two drops of stain on a warm slide and spreading the stain immediately with the aid of a second slide. Viability was assessed by counting 200 sperm cells with bright-field microscopy (×400). Sperm that showed partial or complete colorization were considered non-viable or dead. Only sperm that had strict exclusion of the stain were considered viable.

### Analysis of sperm lipid content

Spermatozoa fatty acid profiles were analyzed at Institute of Standard and Industrial Research of Iran (ISIRI, Karaj, Iran) with the procedures proposed by Safarinejad (3). The fatty acid concentration was determined by GC (0.25×0.32, ID of 0.3 m WCOT Fused Silica Capillary, Agilent 6890, USA) with a 120 m silica-fused column (BPX-70). Nitrogen was the carrier gas. Initial and final temperatures were set at 140 and 240˚C, respectively, with detector and injector temperatures set at 280 and 260˚C. The fatty acid standard was obtained from Sigma-Aldrich. We calculated the desaturase index (monounsaturated fatty acids/saturated fatty acids) for C14, C16 and C18.

### Statistical analysis

Data were analyzed by the t-test using SPSS version 16.0. All values are presented as mean ± SD.

## Results

Live body weight did not differ among CTR and FO groups during the experiment. Except for non-progressive motile sperm, dietary fish oil improved other sperm parameters and semen volume in the feeding phase (phase 1, Table 3). Sperm kinematic parameters (Curvilinear velocity (VCL), VSL (Straight linear velocity) and Average path velocity (VAP); ìm/s) were significantly affected by fish oil inclusion in the feeding phase, whereas the percentage of linearity (LIN) and straightness (STR) sperm as well as wobble (WOB) were unaltered by dietary fish oil.

**Table 3:** Effects of dietary fish oil on mean ram sperm characteristics and sperm kinematic parameters in the feeding phase (phase 1)


	CTR*	FO	SEM

Volume (ml)	1.2^b^	1.5^a^	0.12
Concentration (× 10^9^/ml)	2.70^b^	3.64^a^	0.28
Total sperm output ×)10^9^)	3.38^b^	5.4^a^	0.60
Viability (%)	82^b^	91^a^	3.47
Progressive motility (%)	48^b^	60^a^	5.01
Non-progressive motility (%)	31	31	3.5
Immotile sperm (%)	20^a^	8^b^	2.53
Hyperactive (%)	28.5^b^	33^a^	3.16
LIN** (%)	45	47	2.85
STR (%)	84	83	1.88
WOB (%)	53	55	2.42
VCL (µm⁄ s)	132 ^b^	147 ^a^	9.07
VSL (µm⁄ s)	58 ^b^	68.5^a^	5.55
VAP (µm⁄ s)	69 ^b^	80^a^	5.52


The fatty acid profiles of Zandi ram sperm at commencement of the present study are shown in table 4. C14 (myristic acid), C16 (palmitic acid), C18 (stearic acid), C18:1 (oleic acid) and C22:6 (DHA) were the major fatty acids in the spermatozoa.

At the end of the feeding phase, C14:0, C18:0 and C22:6 n-3 (p<0.01) concentrations increased whereas C6:0, C12:0 (p<0.01) and C18:2 (p=0.07) concentrations decreased with the addition of fish oil to the diet. Other fatty acids were similar between control and FO groups at this stage (Table 4).

**Table 4:** Sperm fatty acid profiles of experimental rams at the commencement of the study and after fish oil consumption in the feeding phase (mean ± SD)


Fatty acid (%)	Before*n=9	After**	
		CTR (n=5)	FO (n=4)

**C6:0**	0.4 ±0.20	5.15±1.3^a^	0.1^b^
**C8:0**	1.65±0.76	0.72±0.2	0.75
**C10:0**	0.8±0.28	4.7±3	0.5
**C12:0**	1.1±0.8	0.7±0.15^a^	0.2 ^b^
**C14:0**	8.5±0.21	6.6±2.7 ^b^	13.2±0.8^a^
**C14:1**	0.3±0.028	0.6	0.45±0.3
**C16:0**	28.7±0.3	22.7±9.1	23.5±3.4
**C16:1**	0.75±0.035	3.5±1.4	1±0.1
**C18:0**	12.6±1.5	6.05±0.87^b^	14.12±0.9^a^
**C18:1**	6.15±0.5	18.06+13	5.5±2.5
**C18:2**	1.25±0.21	7.1±4.4	1.3±0.8
**C20:0**	0.45±0.07	2.7±3.18	0.4
**C18:3n3**	2.63±0.9	4.3±3.4	0.3
**C20:3**	0.35±0.07	2.2±1.7	0.35±0.15
**C22:0**	0.7±0.005	3.07±2.9	1.2±0.1
**C22:1**	3.05±1.4	2.2±1.8	4.05±5.05
**C22:3**	ND	1.8±2.7	0.2
**C22:4**	0.75±0.07	0.54	0.4±0.1
**C22:5**	5.1±0.6	0.5±0.2	0.3±0.1
**C22:6**	14.6±0.84	9.5±1.5^b^	30.8±2.7^a^
**C20:4**	0.3±0.01	3.5±1.1	4.15


Interestingly, the positive effects of fish oil on sperm concentration and total sperm output were observed one and two months after removal of the fatty acid source (p<0.05) whereas other sperm parameters were unaffected (Table 5).

Sperm fatty acid profiles one month after removing the oil source were affected by previous treatment (Table 6). In the FO group, there was an increase in C10:0, C12:0 and C14:0 (p<0.05). The concentrations of C16:1, C20:2 and C18:3 were higher in the CTR group one month after removing the oil source. Sperm DHA (C22:6 n-3) concentration was unaltered between groups (23% of total fatty acids) at this stage.

The concentrations of C8:0, C12:0, C18:0 and C22:6 n-3 were significantly affected by the previous feeding strategy (p<0.05) and they were higher in the FO group compared with the CTR group two months after the oil source was removed (Table 7).

Sperm fatty acid desaturase indices were significantly affected at different sampling times. Before starting diet consumption, this index for C14:1/ C14:0 was 0.035, for C16:1/C16:0 it was 0.026 and for C18:1/C18:0 it was 0.48. Desaturase index increased in CTR group at the end of the first phase, whereas it was constant for the FO group which was the almost same as the start of the experiment.

**Table 5:** Sperm parameters one and two months after removal of fish oil (phase 2) (mean ± SD)


	One month*		Two months*	
Sperm parameters	CTR**	FO	CTR	FO

**Volume (ml)**	1.05±0.1	1.2±0.21	1±0	1.08±0.05
**Concentration (×10^9^/ml)**	1.8±0.19 ^b^	2.7±0.6 ^a^	1.68±0.06 ^b^	2.02±0.17 ^a^
**Total sperm output ×)10^9^)**	1.85±0.28 ^b^	2.9±0.25 ^a^	1.7±0.04 ^b^	2.9±0.0.33 ^a^
**Viability (%)**	76±7.05	83±9.63	78±3.2	83±5.8
**Progressive motility (%)**	51±9.7	59.5±10.04	51.4±5.9	58±4.6
**Non-progressive motility (%)**	23±5.16	23±2.3	23.5±1.3	21.4±2.11
**Immotile sperm (%)**	25±4.8	16.5±9.6	22.2±2.7	19.3±3
**Hyperactive (%)**	30.4±2.5 ^b^	37±4.8 ^a^	31.3±1.2 ^b^	31.6±2.9^b^


**Table 6:** Sperm fatty acid profiles of experimental rams one month after removal of the fish oil (mean ± SD)


Fatty acid (%)	CTR*	FO

**C6:0**	1.3±0.4	0.9±0.1
**C8:0**	0.5±0.2	0.5±0.22
**C10:0**	0.3±0.1^b^	2.7±0.03 ^a^
**C10:1**	0.1±0.08	0.2±0.06
**C12:0**	0.5±0.1^b^	1.65±0.17^a^
**C14:0**	9.5±1.6 ^b^	12.55±0.85 ^a^
**C14:1**	0.4±0.2	ND
**C15:0**	0.7±0.28	ND
**C16:0**	19.7±5.5	20.8±1.5
**C16:1**	1.3±0.2 ^a^	0.6±0.21^b^
**C17:0**	ND	0.4±0.28
**C18:0**	9.13±1.3	8.75±0.8
**C18:1**	11.6±0.45	14.7±2.3
**C18:2**	5.9±2.2	4.35±0.88
**C20:0**	0.2±0	0.25±0.03
**C20:1**	ND	0.45±0.33
**C20:2**	3.8±2.5	0.35±0.035
**C18:3n3**	3.9±2.5	0.35±0.17
**C20:3**	1.3±0.1	1±0.07
**C21:0**	0.3±0.2	ND
**C22:0**	0.2±0.1	1.8±0.91
**C22:1**	3.5±2.8	1.55±0.80
**C22:2**	0.2±0.1	0.2±0
**C22:3**	0.5±0.04	ND
**C22:4**	0.2±0.2	0.1±0
**C22:5**	1.3±0.2	0.3±0
**C22:6**	23.8±3.3	23.55±2.9
**C20:5**	2.6±1.9	0.7±0
**C20:4**	0.5±0	0.4±0
**C24:0**	ND	0.2±0


**Table 7:** Sperm fatty acid profiles of experimental rams two months after removal of the fish oil (mean ± SD)


Fatty acid (%)	CTR*	FO

**C6:0**	4.7±2.5	0.65±0.03
**C8:0**	4±0.24 ^b^	5.2±0.07 ^a^
**C10:0**	3.4±2	0.7±0.1
**C12:0**	4.2±3 ^b^	16.8±0.85 ^a^
**C14:0**	7.9±1.4	8.7±0.1
**C16:0**	17.9±1.4	18.7±0.1
**C18:0**	3.7±1.8 ^b^	6.6±0.3^a^
**C18:1**	12.8±4.9	7.35±0.35
**C18:2**	18.2±9	12.2±4
**C20:3**	0.6	0.5
**C22:6**	11.4±0.4 ^b^	15.3±0.3 ^a^
**C20:5**	1^a^	0.88±0.1^b^


## Discussion

Body weights of all rams remained constant during the experiment. The similar body weight suggested that isoenergetic diets had little impact on energy input and output regulation. In previous studies by Kalkohi and Zandi rams, it was reported that fish oil could be fed up to 35 g/d without an effect on body weight. Thus, constant body weight confirmed that all responses in the present study originated from unique fatty acids in fish oil (2, 9).

As expected, sperm parameters improved dramatically with dietary fish oil in the first phase. The ruminant spermatozoa membranes characteristically contain very high proportions of longchain PUFA, particularly the n-3 series.

Although the mechanisms of the effects of dietary omega-3 fatty acids necessitate additional studies, researchers have proposed their effects on sperm assembly, anti-apoptosis, eicosanoid synthesis and hormone secretion (1). A possible mechanism can be the up/down regulation of some genes’ expressions. It has been assumed that DHA is an obligatory structural component for the formation of some mammalian sperm and it can increase sperm concentration. However, this hypothesis cannot justify improved progressive motility. The n-3 fatty acids accumulate in the tail region of sperm so it is necessary for motility and this improvement may be related to accumulation of DHA in this region. Not only the dietary omega-3 fatty acids have been shown to improve sperm parameters in rams (2, 9) and bulls (15), but a higher intake of omega-3 fatty acids was positively related to sperm morphology in humans (16). Our finding alongside most previous studies showed that dietary omega-3 fatty acids in rams improved sperm parameters when consumed for more than 4 weeks.

Fatty acid profiles in several breeds of species raises the question if this profile is constant between two similar breeds under different managements and diets. Our findings before changing the diet and at the beginning of the study were comparable with those of Samadian et al. (2) (Table 8). Since the rams of two different experiments were all on several diets in different farms, the lack of a dramatic change, exception DHA, in fatty acid profiles between the two studies on Zandi rams has suggested that the major fatty acid profile is an inherent parameter and it may be constant in Zandi rams. The same profiles in a breed of boars have been demonstrated (17).

**Table 8:** Comparison of major sperm fatty acid levels in the current and previous studies in Zandi rams


Fatty acids (% as total fatty acids)	Samadian et al., 2010	Current study

**C14**	7.5	8.5
**C16**	28.5	28.7
**C18**	16	12.6
**C18:1**	7.9	6.1
**C22:6**	22.5	14.6


It appears that consumption of antioxidants as well as dietary fatty acids is the major dietary factor which influences sperm fatty acid profiles. Dietary fats are recognized as a critical item which can influence mammalian spermatozoa and in humans it has been shown that high intake of saturated fatty acids (18) or trans fatty acids (13) may manipulate sperm quality and quantity.

Increased sperm saturated fatty acids is considered as a negative point in normal sperm of humans (18), subfertile men (3, 12) and abnormal boars (14). Surprisingly, in the present study inclusion of fish oil as the dietary polyunsaturated fatty acid source influenced sperm saturated fatty acids. Similarly, Blesbois et al. (19) found fish oil supplementation in roosters’ diet increased sperm C14:0 and sperm quality. Elevation of C14:0 and C18:0 alongside the dramatic increase in DHA concentration suggested that the testes designed sperm fatty acids to maintain membrane fluidity to achieve their biological goal. It has already been established that testes have high capacities for desaturation and elongation of unsaturated fatty acids (1).

Adequate membrane fatty acids mean melting point is necessary for functional performance of biological compounds such as milk (20) and possibly sperm fatty acids (21). In the present study the level of C16:0 between experimental groups was unchanged after the two month feeding. This constant level has also been reported in other studies (7, 21). This consistency may be a part of the scenario of keeping the proper mean melting point during the feeding phase.

Mean melting point (MMP) is proposed as an index of membrane fluidity. Higher MMP by increased saturated or trans fatty acids in oligozoospermia and asthenozoospermia in comparison with normozoospermia corresponds with decreased fluidity of the sperm membrane (21). Therefore it is important to study changes in the major as well as minor fatty acids in sperm after oil consumption. In order to improve sperm quality and quantity in fertile/infertile men or fertile domestic animals it is recommended to consider the testes’ capacity for favorable response. On the other hand, enzymes’ ability and quantity are areas of concern which need to be thoroughly studied in the future.

Interestingly, in the second phase, the positive effects of fish oil on sperm concentration and total sperm output were observed one and two months after removal of the oil source, whereas other sperm parameters were unaffected. In the current investigation, which was the first study (to our knowledge) on sperm parameters one and two months after removal of the dietary oil source, it was surprising to observe the effects of dietary fish oil on some sperm parameters compared with the control. Although it was shown that the nutritional responses have a time lag period and has been recommended that small male ruminants be fed correctly for 2 months before mating (22), our finding suggested that part of this delayed response was compensated after removal of the oil source. Hence, this persistency is important in research farms which use animals in several studies. Further studies are warranted to shed more light on this important issue.

In the second phase, sperm saturated fatty acids were significantly affected by the last feeding strategy, whereas DHA concentrations remained similar one month after removal of the fish oil and a negligible change was observed at two months after removing the oil. For some fatty acids relatively high standard deviations were observed. This variability in fatty acids and higher standard deviations has been demonstrated in previous studies on humans (13) and boars (17), this might have affected our results.

The same concentration of DHA was observed between experimental groups one month after the oil source was removed. Because the antioxidant has a key role in male fertility and sperm function (23), this change in fatty acids and decreased DHA content in the fish oil group in the second phase could be attributed to vitamin E concentrations in the testes, liver and assembled fatty acids in the sperm. In roosters the long-term (34 weeks) consumption of fish oil has been found to drastically deplete the tissues (heart, kidneys, lungs, liver and testes) of vitamin E (24). This reduction in DHA content might be a strategy for maintaining more sperm manifested as elevated sperm concentration and total sperm output. Nevertheless, the effects of dietary fatty acids on sperm fatty acids profile even one and two months after removing fatty acids were observed for the first time.

## Conclusion

The present study showed not only manipulation of ram sperm fatty acid profiles by dietary fish oil and sperm parameters at the feeding period, but also the persistency of unique effects of dietary omega-3 fatty acids effects despite removal of the fat source. Sperm fatty acid profiles are recommended to be analyzed and monitored comprehensively.
